# Effects of Quercetin in a Rat Model of Hemorrhagic Traumatic Shock and Reperfusion

**DOI:** 10.3390/molecules21121739

**Published:** 2016-12-20

**Authors:** Virginia Chamorro, Rachele Pandolfi, Laura Moreno, Bianca Barreira, Andrea Martínez-Ramas, Daniel Morales-Cano, Jesús Ruiz-Cabello, José Angel Lorente, Juan Duarte, Ángel Cogolludo, José Luis Alvarez-Sala, Francisco Perez-Vizcaino

**Affiliations:** 1 Departamento de Farmacología, Facultad de Medicina, Universidad Complutense de Madrid, Madrid 28040, Spain; vchamo26@hotmail.com (V.C.); pandolfi.rachele@gmail.com (R.P.); lmorenog@med.ucm.es (L.M.); danmorca@gmail.com (D.M.-C.); acogolludo@med.ucm.es (Á.C.); 2 Centro de Investigaciones Biomédicas en Red de Enfermedades Respiratorias (Ciberes), Madrid 28029, Spain; biancabarreira@med.ucm.es (B.B.); and.mrt@gmail.com (A.M.-R.); ruizcabe@farm.ucm.es (J.R.-C.); joseangel.lorente@salud.madrid.org (J.A.L.); 3 Instituto de Investigación Sanitaria Gregorio Marañón (IISGM), Madrid 28007, Spain; 4 Centro Nacional de Investigaciones Cardiovasculares Carlos III (CNIC), Melchor Fernández Almagro 3, Madrid 28029, Spain; 5 Servicio de Medicina Intensiva, Hospital Universitario de Getafe, Getafe, Madrid 28905, Spain; 6 Universidad Europea de Madrid, Madrid 28905, Spain; 7 Departamento de Farmacología, Facultad de Farmacia, Universidad de Granada, Granada 18071, Spain; jmduarte@ugr.es; 8 Centro de Investigaciones Biomédicas en Red de Enfermedades Cardiovasculares (Ciberec), Madrid 28029, Spain; 9 Departamento de Medicina, Facultad de Medicina, Universidad Complutense de Madrid, Madrid 28040, Spain; jlasw@separ.es; 10 Servicio de Neumología, Instituto de Investigación Sanitaria San Carlos (IdISSC), Madrid 28040, Spain

**Keywords:** systemic arterial pressure, lung edema, hemorrhage, trauma, reperfusion

## Abstract

Background: We hypothesized that treatment with quercetin could result in improved hemodynamics, lung inflammatory parameters and mortality in a rat model of hemorrhagic shock. Methods: Rats were anesthetized (80 mg/kg ketamine plus 8 mg/kg xylazine i.p.). The protocol included laparotomy for 15 min (trauma), hemorrhagic shock (blood withdrawal to reduce the mean arterial pressure to 35 mmHg) for 75 min and resuscitation by re-infusion of all the shed blood plus lactate Ringer for 90 min. Intravenous quercetin (50 mg/kg) or vehicle were administered during resuscitation. Results: There was a trend for increased survival 84.6% (11/13) in the treated group vs. the shock group 68.4% (13/19, *p* > 0.05 Kaplan–Meier). Quercetin fully prevented the development of lung edema. The activity of aSMase was increased in the shock group compared to the sham group and the quercetin prevented this effect. However, other inflammatory markers such as myeloperoxidase activity, interleukin-6 in plasma or bronchoalveolar fluid were similar in the sham and shock groups. We found no bacterial DNA in plasma in these animals. Conclusions: Quercetin partially prevented the changes in blood pressure and lung injury in shock associated to hemorrhage and reperfusion.

## 1. Introduction

Severe trauma is a leading cause of death and disability worldwide [1]. Hemorrhagic shock after a severe blood loss associated with trauma results in a state of global ischemia. Hemorrhage is managed by fluid resuscitation with crystalloid fluids and blood products which restore the circulating volume and cardiac output [2]. During the reperfusion phase, cell damage induced by ischemia is enhanced due to excessive production of radical oxygen species—a systemic inflammatory response—apoptosis and necrosis, eventually leading to multiple organ failure [3,4]. Thus, global ischemia and the post-reperfusion inflammatory response in hemorrhagic shock are the initial triggering events for the development of acute respiratory distress syndrome (ARDS), a major cause of death in trauma patients. There are no effective treatments that reduce the mortality associated to ARDS, except the hemodynamic and ventilatory support protective strategies [5]. Therefore, the identification of new drugs preventing the ischemia-reperfusion damage could improve survival in patients with trauma.

The flavonoid quercetin is one of the most abundant polyphenolic compounds. It belongs to the subclass of flavonols, is widely distributed in plants and is found naturally in a number of foods in the human diet (including apples, onions, teas, berries, and red wine), in some herbs and it is also available as a dietary supplement. It exerts a wide range of biological actions, including antioxidant, anti-platelet, anti-inflammatory, vasodilator and antihypertensive effects [6,7,8]. Quercetin decreased systemic inflammation and reduced mortality in animal models of lethal endotoxemia [9]. The authors attributed this effect of quercetin to the inhibition of high mobility group box 1 (HMGB1), a crucial nuclear cytokine that elicits severe vascular inflammatory diseases. Activation of acid sphingomyelinase may play a role in endotoxin- and lavage-induced lung injury [10,11] but the effects of quercetin on this signaling pathway are unknown.

Therefore, we hypothesized that quercetin could result in improved hemodynamics, inflammatory parameters and mortality in a rat model of traumatic hemorrhagic shock. The aim of our study is to determine whether the administration of quercetin during resuscitation prevents mortality, systemic and pulmonary inflammatory response and aSMase activation associated to acute lung injury induced by traumatic hemorrhagic shock.

## 2. Results

### 2.1. Mortality

**Figure 1:**
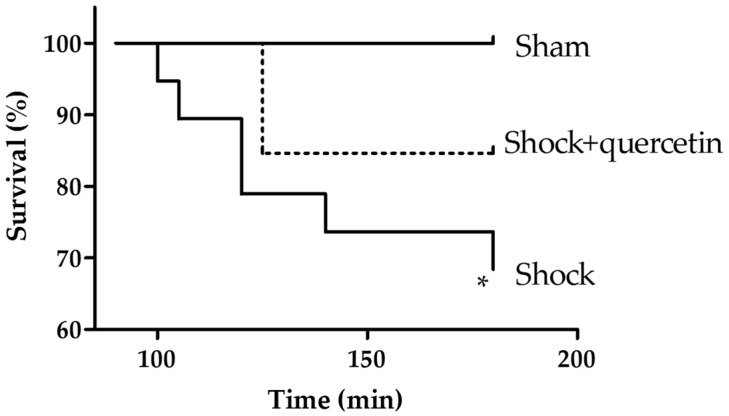
Kaplan–Meier survival analysis during the reperfusion period. Anaesthetized animals were submitted to shock and at time 90 they were reperfused with blood plus lactate Ringer and with vehicle or quercetin, and followed for an additional period of 90 min. * *p* < 0.05 vs. sham (log-rank test).

In the shock group, the survival rate at the end of the reperfusion period was 68.4% (13/19) which was significantly lower than in the sham group (100%). In the treated group (shock + quercetin) the survival rate was 84.6% (11/13) (Figure 1). Despite a favorable trend, the Kaplan–Meier analysis indicated no significant differences in survival between the treated group vs. the shock group.

### 2.2. Arterial Pressure and Heart Rate

**Figure 2:**
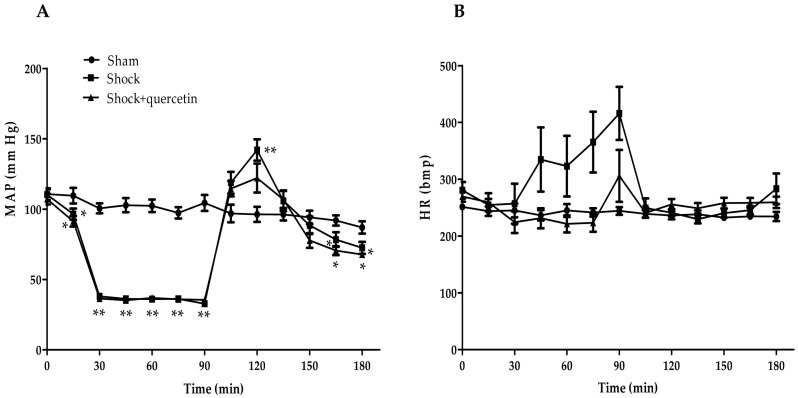
Changes in (**A**) mean arterial blood pressure and (**B**) heart rate in the three experimental groups. Rats were subjected to laparotomy followed by controlled hypotension (shock phase) and then by resuscitation with lactate Ringer solution, shed blood and the quercetin or vehicle. Data are shown as the mean ± S.E.M. * *p* < 0.05 and ** *p* < 0.01 vs. sham group during reperfusion (Dunnett’s test).

Figure 2A shows the changes in the mean arterial pressure in all groups. The laparotomy induced a significant reduction of the arterial pressure (at 15 min) and a sustained hypotension (shock phase) was achieved by bleeding the animals to a target pressure of 35 mmHg (from 15 to 90 min). The reperfusion produced an initial transient hypertension during the reinfusion period of the blood plus lactate Ringer, followed by a hypotensive phase. The hypertensive phase was less marked in the animals treated with quercetin. Figure 2B shows that during the shock period, an increase in heart rate was observed in some animals but, taken together, differences were not statistically different among groups. In the reperfusion phase, heart rate was similar in all groups.

### 2.3. Acid Sphingomyelinase Activity

**Figure 3:**
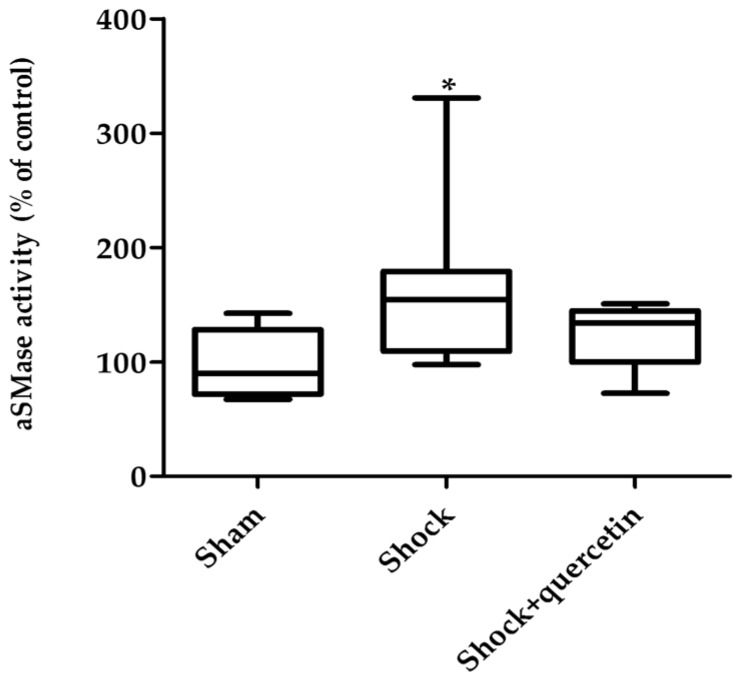
aSMase activity. Measurements were made in lung homogenate for a total time of 30 min expressed as nmol/min/mL. * *p* < 0.05 vs. sham group. Box-and-whisker plot showing medians with interquartile range and minimum and maximum values.

At the end of the reperfusion period, aSMase activity was increased in lung homogenates from the animals exposed to traumatic shock and resuscitation (Figure 3). Quercetin prevented the activation of the enzyme.

### 2.4. Blood Gases

**Figure 4:**
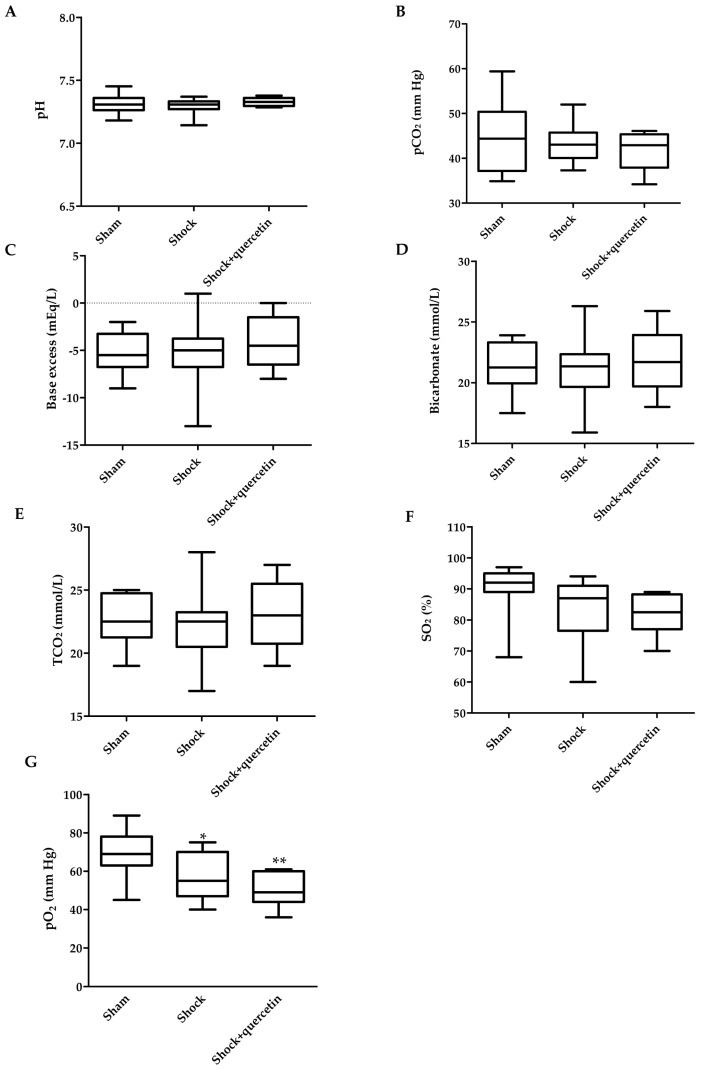
Arterial blood gases. (**A**) Values of pH; (**B**) carbon dioxide partial pressure (pCO_2_); (**C**) excess base; (**D**) bicarbonate (HCO_3_^−^); (**E**) total carbon dioxide concentration (TCO_2_); (**F**) arterial oxygen saturation (SO_2_) and (**G**) arterial oxygen partial pressure (pO_2_) in the sham, shock and shock + quercetin at 180 min (at the end of reperfusion). Box-and-whisker plot showing medians with interquartile range and minimum and maximum values. * *p* < 0.05 and ** *p* < 0.01 vs. sham group.

After 90 min of shock, animals developed metabolic acidosis with a decrease in pH (7.19 ± 0.02 vs. 7.32 ± 0.02 in the sham group), excess base (−10.5 ± 1.2 vs. −2.8 ± 1.3 mEq/L), bicarbonate (17.2 ± 1.2 vs. 23.3 ± 1.2 mmol/L), TCO_2_ (18.4 ± 1.3 vs. 24.5 ± 1.1 mmol/L) (*p* < 0.05 for all parameters, Student’s *t* test) without changes in SO_2_ and pCO_2_. At the end of the reperfusion period, the values of pH, pCO_2_, base excess, bicarbonate and TCO_2_ in the shock group were restored (*p* > 0.05 vs. the sham group, Figure 4A–E) but animals developed hypoxemia with a significant decrease in pO_2_ and a similar trend for SO_2_ (Figure 4F,G). The hypoxemia induced by shock and reperfusion was not reverted by quercetin.

### 2.5. Electrolytes, Hematocrit and Hemoglobin

At the end of shock phase, an increase in plasma potassium and a moderate decrease in sodium were observed in the shock group with no changes in plasma calcium. Anemia, resulting from hemorrhage during the shock phase, was reverted after the reinfusion period. Electrolyte, hematocrit and hemoglobin values at the end of reperfusion were similar in all groups (Table 1).

### 2.6. Markers of Inflammation, Pulmonary Vascular Permeability, Histological Changes and Bacterial Translocation

**Figure 5:**
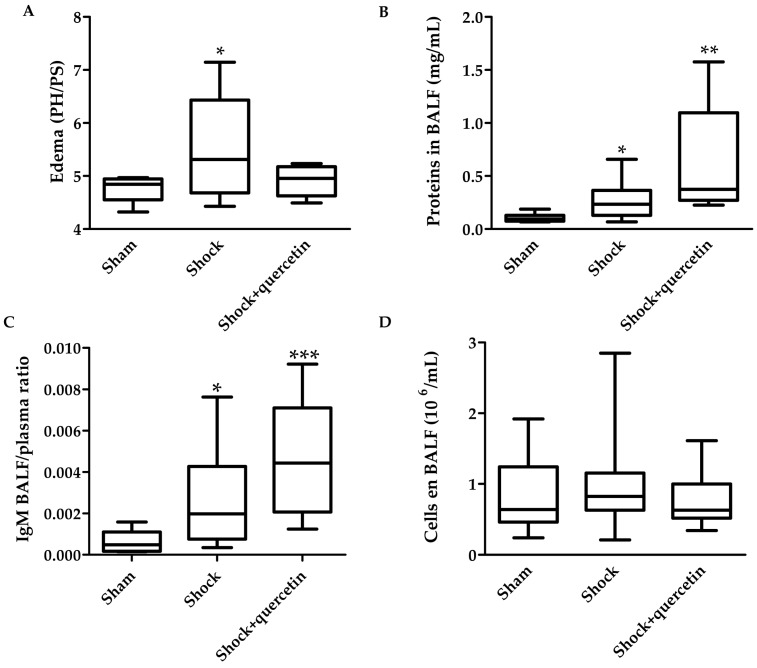

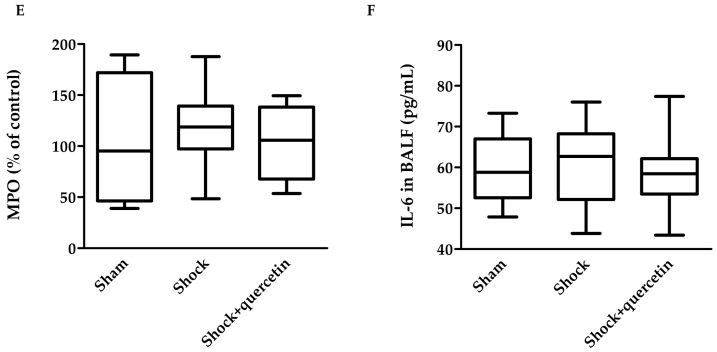
Markers of inflammation and vascular permeability: (**A**) Edema (wet to dry weight ratio); (**B**) proteins in Bronchoalveolar Lavage Fluid (BALF); (**C**) IgM BALF/plasma ratio (**D**) cells in BALF; (**E**) Myeloperoxidase (MPO) activity and (**F**) IL-6 in BALF measured at the end of reperfusion. Box-and-whisker plot showing medians with interquartile range and minimum and maximum values in each group. * *p* < 0.05, ** *p* < 0.01 and *** *p* < 0.005 vs. sham group.

Figure 5A shows that animals submitted to shock plus reperfusion develop pulmonary edema, as indicated by an increase in the wet to dry lung weight ratio. Quercetin inhibited the development of edema. Proteins in bronchoalveolar lavage fluid (BALF) and IgM, used as markers of pulmonary vascular permeability, increased in the shock compared to the sham group (Figure 5B,C). Paradoxically, quercetin increased the proteins levels and IgM concentration in BALF. Cell counting in BALF and the myeloperoxidase activity in lung homogenates were not significant among groups (Figure 5D,E). IL-6 concentration in BALF (Figure 5F) or in plasma (not shown) was similar between sham and shock groups. 

To analyze bacterial translocation, DNA in plasma was determined using specific primers for conserved regions of bacterial 16S gene. We found no bacterial DNA in plasma in any groups, indicating the lack of bacterial translocation.

## 3. Discussion

Reperfusion-induced organ damage after hemorrhagic shock is associated to oxidative stress and a systemic inflammatory status, resulting in a significant mortality. At present, there is no effective treatment for this condition. Herein, we show that quercetin prevented partially the reperfusion damage in a rat model of hemorrhagic traumatic shock. 

Several hemorrhagic shock-resuscitation models have been established but none of them covers all of the important aspects related to clinical reality [12,13,14]. A short period of severe hypotension Mean Arterial Pressure (MAP) lower than 25 to 35 mmHg for only 20 min does not result in life-threatening organ injury and it is not clinically relevant [15]. Our experimental schedule of trauma induction within 15 min, a hemorrhagic shock period of 75 min and subsequent resuscitation for 20–30 min, is orientated toward human clinical practice and reflects most emergency situations realistically [16]. The model was associated, at the end of the reperfusion period, with hypotension, hypoxemia, lung edema, increased pulmonary vascular permeability and 30% mortality. However, we found little changes in inflammatory markers. The protocol of drug administration at the beginning of the resuscitation was also clinically oriented in order to analyze the potential effects to prevent reperfusion-induced damage at the Intensive Care Unit (ICU).

Rats receiving shed blood plus the same volume of Ringer’s lactate with quercetin during resuscitation restored the blood pressure after 20–30 min. Reperfusion produced an initial transient hypertension during the reinfusion period of the blood plus lactate Ringer. This was followed by the characteristic features of shock with associated organ injury, a hypotensive phase and hypoxemia. Hyponatremia, hyperpotassemia and anemia were recovered by the fluid and blood re-infusion. Quercetin prevented the hypertensive phase but did not correct the hypoxemia and hypotension induced by shock and reperfusion. The mortality in the shock vehicle group was 30% and a trend for increased survival in the quercetin treated group (15%) was observed. 

The pulmonary edema was shown by an increase in the wet to dry lung weight ratio in the shock group and this was accompanied by increased total proteins in BALF. Nevertheless, inflammation markers such as myeloperoxidase, IL-6 in BALF and in plasma and the cells infiltration in BALF were similar in the shock and sham groups. Remarkably, quercetin prevented the formation of edema. Paradoxically, the changes in total proteins and IgM in BALF were not prevented by quercetin. Flavonoids were initially described to be protective on the permeability of vascular capillaries as early as the 1930s [17]. The fact that quercetin reduced edema but not the change in proteins or IgM suggests that the anti-edema effect is due to reduced hydrostatic pressure rather than a direct effect on vascular permeability. This is consistent with the prevention of the hypertensive phase by quercetin during blood and fluid administration which is presumably accompanied by reduced pulmonary arterial pressure.

Sphingolipids are structural components of eukaryotic cellular membranes [18] whose hydrolysis products (ceramide, sphingosine and sphingosine-1-phosphate, S1P) play a key role in signalling pathways involved in different cellular processes, such as cell growth and differentiation, inflammation, apoptosis and vascular tone [19]. Alteration of the sphingolipid signaling pathway may play a pathophysiological role in several conditions, such as diabetes, atherosclerosis, chronic heart failure, sepsis, cancer and several lung diseases including ARDS, cystic fibrosis and Chronic Obstructive Pulmonary Disease (COPD) [18,20,21]. aSMase is a critical enzyme in sphingolipid biochemistry that, under conditions of inflammation or stress, can rapidly be activated to convert sphingomyelin to ceramide [22]. A well described consequence of aSMase activation is formation of ceramide-rich membrane microdomains which are involved in several cell functions, such as stress signalling, apoptosis after death cell receptor stimulation and infection with various pathogens [18,22]. 

These findings suggest that sphingolipids should be studied further as a therapeutic target in ARDS [21]. Activation of aSMase and increased ceramide levels has been reported to play a role in acute lung injury induced by endotoxin [10], Platelet Activating Factor (PAF) [23] and by surfactant washout [11]. Herein, we report for the first time increased aSMase activity in lung homogenates following traumatic hemorrhagic shock and reperfusion, thus confirming that aSMase activation is a common feature of animal models of acute lung damage. Moreover, aSMase inhibition prevented acute lung injury caused by PAF [23] or by repeated airway lavage [11]. We also found that quercetin prevented the increase in lung aSMase activity. Therefore, this suggests that aSMase inhibition is a novel potential mechanism of organ protection by quercetin.

## 4. Materials and Methods

### 4.1. Animals

The investigation conforms to the *Guide for the Care and Use of Laboratory Animals* published by the US National Institutes of Health (publication No. 85–23, [24]), and the procedures were approved by our institutional Ethical Committee. Male Wistar rats (*n* = 44; weight, 275 to 325 g; age, 12 weeks) were obtained from Harlan Laboratories (Barcelona, Spain). Animals were kept under standard conditions of temperature 22 ± 1 °C) and 12:12 h dark/light cycle with free access to food and water. 

### 4.2. Anesthesia, Pressure Measurements and Blood Gases

Rats were anesthetized (80 mg/kg ketamine plus 8 mg/kg xylazine i.p.) and the carotid artery and internal jugular vein were isolated and cannulated with polyethylene catheters (PE-50). Despite ketamine being reported to induce disparate antioxidant/prooxidant effects depending on the dose and organs [25], the anti-oxidant effect of quercetin has been widely reported in animals anaesthetized with the anesthetic mixture (e.g., [26]). Both catheters remained in place for the duration of the experiment and were fixed with surgical suture (skin). Animals remained anesthetized throughout the duration of the experiment by continuous intravenous infusion of ketamine plus xylazine (60 and 3 μg/kg/min, respectively). Systolic, diastolic, mean systemic arterial pressures and heart rate were analyzed with a pressure transducer via a catheter located in the carotid artery in anesthetized rats. Blood samples were collected from the carotid artery. Arterial oxygen saturation (SO_2_) was continuously recorded by a pulsioxymeter (StarrOx) placed in the paw of the animal. Arterial oxygen and carbon dioxide partial pressure (pO_2_ and pCO_2_, respectively), SO_2_, pH, acid-base status, hemoglobin concentration, hematocrit, electrolytes (Na^+^, K^+^, Ca^2+^), and glucose were assessed by using a blood gas analyzer (i-Stat, Softonic, Abbott, Madrid, Spain).

### 4.3. Study Groups and Protocol

Animals were randomly allocated to three experimental groups (*n* = 11–20 in each one): (1) sham group plus vehicle (100 μL DMSO, sham); (2) shock plus vehicle (shock); (3) shock + quercetin (50 mg/kg). The temperature of the animals was maintained at approximately 37 °C, using an electric heating pad under the surgical platform. After surgery, rats were stabilized for 10 min. Rats in the sham group were followed for 180 min and vehicle was administered at time 90 min (100 μL DMSO infused manually over a period of 5 min). Rats in the shock groups were submitted to a protocol of trauma plus hemorrhagic shock followed by resuscitation [26]. Briefly, a 3 cm midline laparotomy was performed with exposure of the intestine for 15 min (trauma), followed by reintroduction and closure with a 4–0 silk suture. Hemorrhagic shock was induced by blood withdrawal in order to reduce the mean arterial pressure to 35 mmHg and maintained at this level for 75 min by withdrawing or re-infusing blood as needed. At the end of the hemorrhagic shock (time 90 min), the animals were resuscitated by re-infusion of all the shed blood plus the same volume of lactate Ringer (over a period of approx. 20–30 min) restoring the blood pressure [27,28]. Quercetin was administered intravenously (50 mg/kg). Eventually, the time of death was recorded for survival analysis. Blood samples were taken from the carotid artery catheter at time 15 (after trauma), 90 (at the end of the shock period) and 180 min (end of the experiment). Blood plasma was obtained by centrifugation (1000 rpm, 15 min, RT) and stored at −80 °C until use. At the end of the experiment, surviving animals were sacrificed by a tracheotomy and thoracotomy performed by midline incision and the right bronchium was ligated. Ten mL cold saline solution (0.9% NaCl, 2.5 mL four times) was administered intratracheally; then, 7–8 mL of bronchoalveolar lavage fluid (BALF) was collected and centrifuged (1500 rpm, 10 min, RT) for the determination of the following parameters: cell number (10^6^/mL) and phenotype, lung permeability, protein (mg/mL), interleukin-6 (IL-6) (pg/mL) and immunoglobulin M (IgM) concentration. In a subset of the animals, two lobes of the right lung were collected and placed in liquid nitrogen until storage at −80 °C to analyze changes in lung vascular permeability and to measure the activity of the acid sphingomyelinase and myeloperoxidase enzymes as markers of the inflammatory process. The other lobe of the right lung was weighed and put in a laboratory oven at 50 °C for 24 h in order to determine pulmonary edema by calculating the lung wet-to-dry (W/D) weight ratio (W/D). In the other subset, the lungs were used for histopathologic evaluation. 

### 4.4. Myeloperoxidase Activity Assay (MPO)

MPO activity was measured in frozen lung tissue, homogenized in pestle and mortar with liquid nitrogen and then with a pellet pestle motor for 30 s (0.1 g of lung/500 μL phosphate buffer) and centrifuged (20 min, 13,000 rpm at 4 °C). The pellet was resuspended in 300 μL of phosphate buffer containing 0.5% hexadecyltrimethylammonium bromide (CTAB) and subjected to three cycles of sonication, freezing and thawing prior to a final centrifugation step. The supernatant generated was assayed for MPO activity using kinetic readings over 12 min at 450 nm (10 μL sample with 100 μL solution containing 0.167 mg·mL^−1^ of *O*-dianisidine dihydrochloride and 0.0006% H_2_O_2_). MPO activity was evaluated in 4 μg of protein for each sample and expressed as MPO/min/μg of proteins.

### 4.5. Acid Sphingomyelinase Assay (aSMase)

Acid sphingomyelinase activity was measured using a commercial fluorimetric assay kit (Cayman). Briefly, aSMase was determined in lung homogenate using an aSMase solution provided by the kit (50 mM sodium acetate, pH = 5; 0.01 g of lung/100 μL aSMase solution). After incubation on ice for 15 min, the homogenate was centrifuged (2780 rpm, 4 °C, 10 min). The supernatant was collected and sonicated (5 s) and aSMase activity was analyzed using the fluorometer Varioskan (Thermo Scientific, excitation and emission wavelengths of 535 and 590 nm, respectively). The activity was determined in 36 μg protein and expressed as nmol/min/mL [11].

### 4.6. Bacterial Translocation

DNA was isolated from plasma (200 μL) using a QIAamp DNA Mini Kit (Qiagen, Hilden, Germany) according to the manufacturer’s instructions and amplified by quantitative RT-PCR using a Taqman system (Roche-Applied Biosystems, Mennheim, Germany) in the Genomic Unit of University Complutense of Madrid. Specific primers and a probe were designed for the conserved regions of bacterial 16S rDNA: the forward primer, 5′-TCCTACGGGAGGCAGCAGT-3′, the reverse primer 5′GGACTACCAGGGTATCTA ATCCTGTT-3′ and the probe (6-FAM)-5′-CGTATTACCGCGGCTGCTGGCAC-3′-(TAMRA) [29]. *E. coli* DNA was used as a positive control.

### 4.7. Cytokine and IgM Assay

IL-6 and IgM concentrations were determined in plasma (dilution 1:10 and dilution 1:2000 respectively) and in BALF (dilution 1:5 for IgM) using ELISA Kits (DuoSet ELISA Development Systems-Research and Development System, Rat IgM Ready-Set-go, Affimetrix).

### 4.8. Statistical Analysis

Results are expressed as means ± SEM of measurements or as medians with interquartile range and minimum and maximum values in a box-and-whisker plot. Survival graphs were constructed according to the Kaplan–Meier method, and the log-rank test was used to compare curves. For normally distributed data, statistical analysis was performed by one-way ANOVA and subsequent Dunnett’s post hoc test (except for two sample comparisons at time 90 min which were analyzed by a Student’s *t* test). Otherwise, a non-parametric Kruskal–Wallis test followed by Dunn’s multiple comparison test was used. A value of *p* < 0.05 was considered statistically significant. 

## 5. Conclusions

In conclusion, quercetin presents a partial protective effect in this shock–trauma model, preventing the activation of aSMase and inhibiting the development of edema. Moreover the hypertensive phase was less marked in the animals treated with quercetin. Given the lack of effective treatments for this severe condition, together with a favorable safety profile of this flavonoid, we suggest that quercetin might be useful in patients at risk of ischaemia reperfusion injury following reperfusion in patients suffering a hemorrhagic shock.

## Figures and Tables

**Table 1:** Electrolytes, hematocrit and hemoglobin (Hb).

Group	Shock Phase (*t* = 90 min)					Reperfusion (*t* = 180 min)				
	Na	K	Ca	Hb	Hematocrit	Na	K	Ca	Hb	Hematocrit
Sham	137.4 ± 0.82	5.30 ± 0.13	1.28 ± 0.05	13.38 ± 0.37	39.40 ± 1.07	137.0 ± 2.84	5.10 ± 0.16	1.19 ± 0.11	12.62 ± 0.33	37.11 ± 0.95
Shock	132.0 ** ± 0.85	6.33 ** ± 0.26	1.18 ± 0.12	9.50 ** ± 0.50	27.88 ** ± 1.47	131.79 ± 2.37	5.84 ± 0.17	0.95 ± 0.09	11.59 ± 0.37	34.07 ± 1.09
Shock + quercetin	nd	nd	nd	nd	nd	133.36 ± 1.52	5.40 ± 0.17	1.18 ± 0.12	12.93 ± 0.42	38.00 ± 1.22

** *p* < 0.01 vs. sham (Student’s *t* test). The one-way Anova indicated no significant differences during reperfusion.
